# Hyaluronic acid-based osmolality-responsive immediate-release microneedles co-loading baicalein and astaxanthin for oral ulcer therapy

**DOI:** 10.1016/j.mtbio.2026.103702

**Published:** 2026-10-01

**Authors:** Jinfan Yang, Kaihong Shi, Jiachen Wang, Huimin Liu, Yang Zhang, Xiaoyu Guang, Siyuan Wang, Wenxi Lei, Yuxiao Wang, Mingqian Tan, Xiaobin Ma, Hao Wu

**Affiliations:** a Comprehensive Breast Center, The Second Affiliated Hospital of Xi'an Jiaotong University, Xi'an, 710061, China; b National Demonstration Center for Experimental Light Chemistry Engineering Education, Shaanxi Provincial Key Laboratory of Papermaking Technology and Specialty Paper Development, College of Bioresources Chemical and Materials Engineering, Shaanxi University of Science & Technology, Xi'an, 710021, China; c School of Basic Medical Sciences, Key Laboratory of Environment and Genes Related to Diseases of Ministry of Education, Xi'an Jiaotong University, Xi'an, 710061, China; d Academy of Food Interdisciplinary Science, School of Food Science and Technology, Dalian Polytechnic University, Qinggongyuan1, Ganjingzi District, Dalian, 116034, China

**Keywords:** Responsive microneedles, Immediate release, Hyaluronic acid, Oral ulcers, Baicalein, Astaxanthin

## Abstract

Oral ulcers, a prevalent mucosal disorder, are clinically managed by topical corticosteroids and immunosuppressants to alleviate symptoms. However, conventional therapies suffer from suboptimal drug bioavailability, inconsistent efficacy, and potential adverse effects. To address these limitations, using natural carbohydrate polymer hyaluronic acid (HA) with excellent biocompatibility, as the main substrate, we constructed the osmolality-responsive immediate-release microneedles (MNs) platform (GA@BA&Ast MNs) co-loading baicalein (BA) and astaxanthin (Ast) for oral ulcer therapy, which effectively addresses traditional treatment bottlenecks via responsive immediate-release and efficient absorption. The platform's core innovation lies in its dynamically controllable release mechanism, which is triggered specifically by the oral microenvironment. Its osmolality-responsive mechanism, relying on HA-Ca^2+^ dynamic coordination network dissociation in oral fluid, enables rapid disintegration and delivery speed superior to conventional microneedles, with good mechanical strength for irritation-free mucosal penetration and 85.3%/87.1% cumulative BA/Ast release within 20 min. In the rat model, GA@BA&Ast MNs significantly accelerated ulcer healing, increased collagen deposition and VEGF expression by 41.22% and 47.05%, and exerted complementary antibacterial, anti-inflammatory, and ROS scavenging effects by promoting epithelial migration and angiogenesis. This responsive MNs platform overcomes the limitations of hydrophobic drug delivery, providing a promising universal strategy for localized orofacial diseases, such as oral ulcers, oral fibrosis, and oral cancer.

## Graphical abstract

**Figure:**
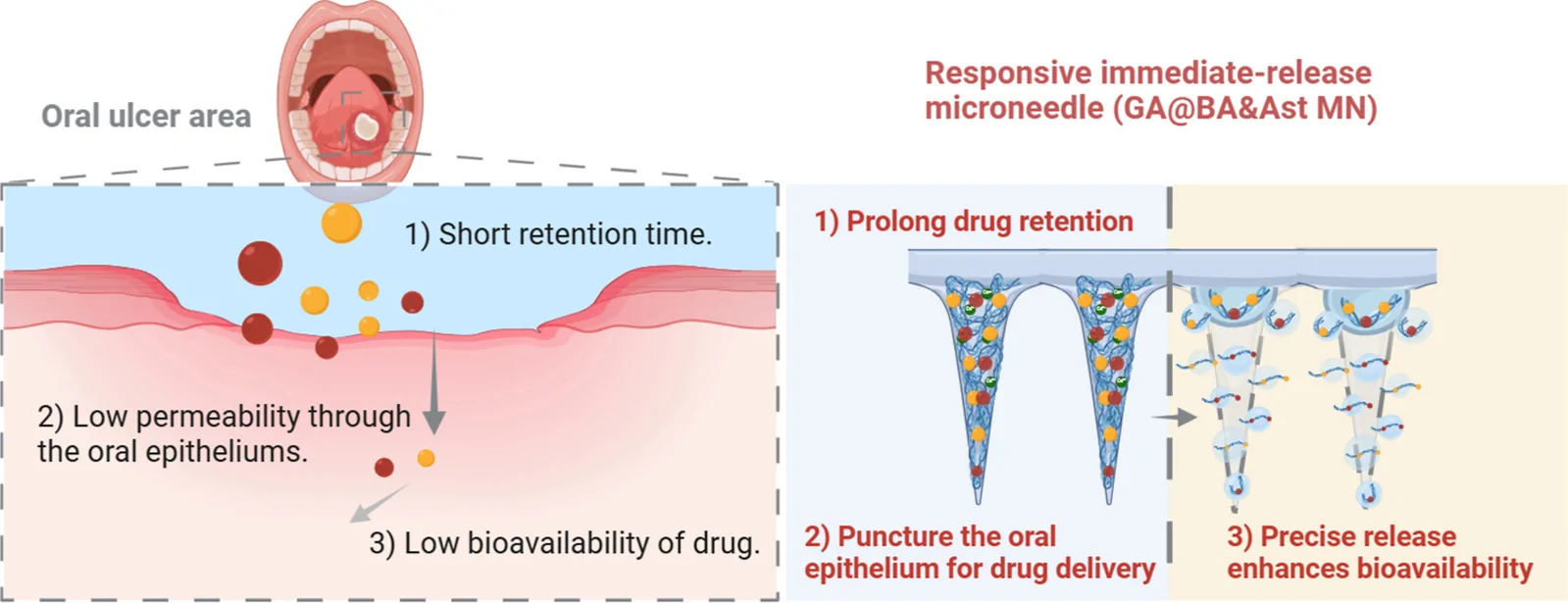

## 1 Introduction

The oral mucosa is part of the human nonspecific immune barrier. Oral mucosal drug delivery for treating diseases such as oral ulcers, oral fibrosis and oral cancer has many limitations due to the oral environment [[1], [2], [3], [4]]. The main challenges are: 1) short retention time of drugs due to continuous saliva wash-away and swallowing reflex; 2) extremely low permeability of hydrophilic drugs through the oral epithelium because of the stratum corneum barrier; and 3) low bioavailability of released drugs in the oral epithelium [[5], [6], [7], [8]].

To address these challenges, currently applied localized oral drug delivery technologies, such as soluble adhesive patches, can partially reduce the effects of saliva wash-away [9,10]. However, it remains difficult to overcome the stratum corneum barrier and enzymatic degradation, which often prevent ideal therapeutic outcomes due to inefficient drug delivery [[11], [12], [13]]. Drug-loaded microneedles (MNs) provide a promising solution. By physically penetrating the mucosa and releasing drugs into the submucosal compartment, MNs effectively avoid the low mucosal permeability associated with crossing the stratum corneum barrier. They also partially escape rapid clearance caused by saliva wash-away [14,15]. However, disruption of the local barrier by MNs may also facilitate diffusion of drugs back into the oral cavity, exhibiting-concentration dependent diffusion effects [16]. In addition, minor tissue damage caused by MNs insertion and residual MNs fragments retained within the epithelial tissue can persistently induce localized inflammatory responses, leading to an accumulation of inflammatory cytokines from immune infiltration, which can complicate the healing process of oral ulcers and other conditions [17]. Water-soluble MNs whose structures dissolve and clear away in saliva and tissue fluids can release drugs while potentially mitigating inflammatory reactions [18].

However, to ensure sufficient mechanical strengths, soluble MNs often depend on macromolecules as the backbone structure [19]. The dissolution kinetics of these MNs can range from several hours to days, which incidentally prolong both drug release and duration of inflammation response that is unfavorable for tissue healing [20,21]. Therefore, constructing a system capable of responsive and controllable decomposition in epithelial tissues for on-demand rapid drug release coupled with immediate drug uptake by the tissue would be highly desirable as an ideal strategy for oral mucosal drug delivery [22].

Current mucoadhesive microneedle systems are mainly fabricated using slowly degraded polymers (such as poly(lactic-co-glycolic acid) (PLGA) and chitosan (CA)) or cross-linked hydrogels (such as HA and gelatin) [23,24]. Their degradation/release profiles are determined by the material properties and local tissue conditions. Although these systems enable sustained drug action, they fail to achieve adequate initial burst release (<30% loading dose) that is essential for early suppression of acute inflammation [25]. In addition, residual microneedle fragments or undegraded polymer matrix could trigger foreign body response, resulting in a vicious cycle of “secondary injury-delayed repair”. For example, caprolactone-based microneedles undergo incomplete hydrolysis in wet oral cavity, generating microparticles that stimulate neutrophils to produce excessive ROS, thereby exacerbating ulcer oxidative stress [26,27].

To achieve this, we designed a soluble drug-loaded microneedle with a tunable, osmolality-responsive immediate-release mechanism for oral epithelia (Fig. 1). The design is based on self-assembled long-range orderly drug-loaded nanofibers by mimicking the triggered-release strategy of nematocyst release from jellyfish sting cells in response to osmotic pressure [28]. The objectives are: 1) to achieve controlled immediate-release of > 85% anti-inflammatory load within 20 min post insertion through a triggered dissolution process, together with fast clearance of the microneedle construct to eliminate early inflammation cascade; and 2) to take advantage of biomimetic immediate-release drug-loaded nanofibers that feature a quick uptake by epithelial cells to overcome the problem of short retention time of drugs and significantly reduce the risk of enzymatic degradation for sustained [29,30] and highly efficient local drug utilization in the oral cavity [31].

**Fig. 1:**
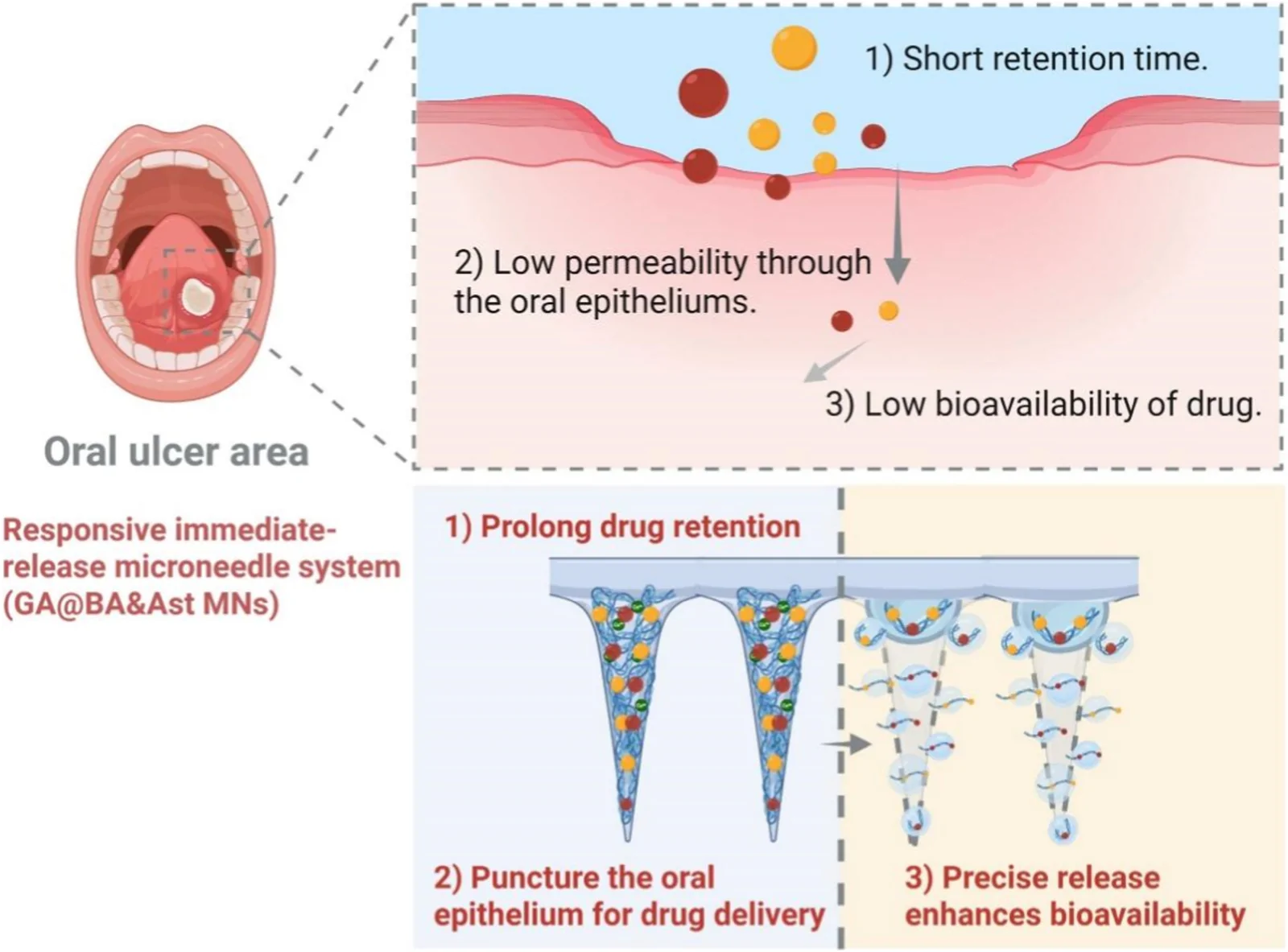
Responsive immediate-release mechanistic action of GA@BA&Ast MNs against Oral ulcer.

A dynamically switchable coordination network forms the basis of this responsive system. HA, with its abundant carboxyl groups, can not only react with multivalent cations (e.g., Ca^2+^/Zn^2+^) but also spontaneously form a three-dimensional hydrogel network via hydrogen bonds in a humid environment [[32], [33], [34]]. When the gel encounters tissue fluid exudates (especially Na^+^/K^+^), ligand exchange displaces prechelated multivalent cations and induces rapid dissociation of the gel, especially for low molecular weight HA [35]. Relevant studies have clearly confirmed that the dissolution efficiency of HA gel could be more than five times higher than that of chitosan gel [36]. Therefore, the backbone structure of the water-soluble drug-loaded microneedles was selected as the gel system formed by low molecular weight HA and Ca^2+^, provided it ensured that the microneedles had sufficient mechanical strength to penetrate the oral epithelium [37].

To mimic the jellyfish stinging cell-inspired bionic release system, we constructed drug-loaded nanofibers GA@BA and GA@Ast based on the internal hydrophobic domains of long-range ordered nanofibers formed by glycyrrhizic acid (GA) self-assembly [38,39]. These nanofibers respectively load baicalein (BA) and astaxanthin (Ast), which possess complementary therapeutic properties. BA functions by disrupting bacterial cell walls and inhibiting DNA synthesis to exert antibacterial effects, while Ast excels at scavenging reactive oxygen species (ROS) [40,41] and promoting tissue repair, thereby facilitating epithelial healing and restoration [42,43]. In addition, the structural characteristics of the drug-loaded nanofibers and their abundant surface carboxyl groups endow them with properties like anionic polymers. This study demonstrated that these drug-loaded nanofibers can also form gels with Ca^2+^ and be integrated into the HA gel network, thereby achieving a jellyfish stinging cell-mimetic release strategy [44] targeting key mechanisms of ulcer pathogenesis to achieve anti-inflammatory, antibacterial, and redox homeostasis regulation effects, while avoiding corticosteroid-related side effects [45].

In this study, to achieve the responsive and controllable immediate release of drugs, two drug-loaded nanofibers (GA@BA and GA@Ast) were prepared using low-frequency acoustic resonance technology [46]. A composite microneedle platform (GA@BA&Ast MNs) was fabricated by templating a coordination-mediated hydrogel network formed through coordination between selectively chosen low molecular weight HA, drug-loaded nanofibers, and calcium ions. It is expected that after insertion into the oral cavity epithelium at the site of the ulcer, the microneedles will rapidly and responsively release the drug-loaded nanofibers (GA@BA and GA@Ast) [47], which are then readily taken up by surrounding epithelial cells, thereby achieving precise drug delivery within the oral cavity epithelium [48]. This strategy aims to exert complementary anti-inflammatory, antibacterial, and redox homeostasis regulation effects, thus accelerating the healing of oral ulcers [49,50].

we hypothesize that by constructing a dynamic and tunable coordination network, and based on the long-range ordered nanofibers with internal hydrophobic domains formed by the self-assembly of GA, drug-loaded nanofibers GA@BA and GA@Ast are fabricated. This serves to prepare a triggerable, bionic release system based on a MN platform. This platform combines the mechanical strength suitable for penetration with the ability to rapidly disintegrate in response to tissue osmotic pressure. It overcomes the inherent poor aqueous solubility and low bioavailability of phytochemical-derived therapies, achieving responsive bionic immediate release and efficient absorption, thereby effectively traversing the mucosal barrier, and enhancing the local bioavailability of the drugs. Through the complementary effects of anti-inflammatory, antibacterial, and antioxidant mechanisms, it is anticipated to accelerate the healing of oral ulcers.

## 2 Materials and methods

### 2.1 Materials and reagents

GA (purity ≥98%, CAS: 1405-86-3) and BA (purity ≥98%, CAS: 491-67-8) were purchased from Shanghai Aladdin Biochemical Technology Co., Ltd. Ast (purity ≥98%, CAS: 472-61-7) was obtained from Shanghai Yuanye Bio-Technology Co., Ltd. HA(Mw: 585097 Da, PDI: 2.130663, CAS: 9004-61-9) (Fig. S1) was acquired from Shanghai Macklin Biochemical Co., Ltd. Polydimethylsiloxane (PDMS) microneedle molds were sourced from Suzhou Zhongke Qiheng Scientific Instrument Co., Ltd., with specifications including 225 (15×15) needle tips, a needle height of 600 μm, an inter-needle spacing of 600 μm, a base diameter of 300 μm, and a patch substrate size of 10 × 10 mm. Enzyme-linked immunosorbent assay (ELISA) kits for interleukin-6 (IL-6) and tumor necrosis factor-α (TNF-α) detection were purchased from Hangzhou Lianke Biotechnology Co., Ltd. The reactive oxygen species (ROS) detection reagent (DCFH-DA) was procured from Fande (Beijing) Biotechnology Co., Ltd. All other chemicals were of analytical grade, and ultrapure water was used throughout the experiments.

### 2.2 Characterization of hyaluronic acid

The chemical structure of the hyaluronic acid raw material was confirmed by proton nuclear magnetic resonance (^1^H NMR) spectroscopy. The sample was dissolved in deuterium oxide (D_2_O) at a concentration of 5 mg mL^−1^, and the spectrum was recorded at 25°C using a Bruker 600 MHz spectrometer. The molecular weight and its distribution were determined by gel permeation chromatography (GPC). The GPC system was equipped with a refractive index detector and an appropriate chromatographic column. A 0.1 M NaNO_3_ solution was used as the mobile phase at a flow rate of 0.6 mL min^−1^, the column temperature was maintained at 40°C, and the sample concentration was 2 mg mL^−1^. The molecular weight was calculated based on an established calibration curve.

### 2.3 Preparation of GA@BA and GA@Ast nanofiber hydrogels

Drug nanofiber complexes were prepared using low-frequency acoustic resonance self-assembly technology. Specifically, GA and BA were co-dissolved in ultrapure water at the predetermined mass ratio and subjected to low-frequency acoustic resonance treatment at 55 Hz with an acceleration of 50 g for 30 min. This physical-field-assisted process promoted molecular ordering and self-assembly, yielding a homogeneous and stable GA@BA nanofiber suspension. GA@Ast nanofibers were prepared using the same procedure and processing.

The GA was accurately weighed with BA and Ast according to predetermined optimized mass ratios (GA/BA = 20:1, GA/Ast = 15:1), respectively, and co-dissolved in ultrapure water. The mixed solution was then treated for 30 min in a low-frequency acoustic resonance processing system to form uniform GA@BA and GA@Ast nanofiber suspensions. To induce gelation, 20 μL of CaCl_2_ aqueous solution (11.1 mg×mL^−1^) was slowly added dropwise to 1 mL of the nanofiber suspension. The mixture was shaken upside down and left to stand for 30 s to obtain the final GA@BA and GA@Ast hydrogels.

### 2.4 Time-dependent cellular uptake and lysosomal co-localization

Cy5-loaded GA nanofibers (GA@Cy5) were prepared using low-frequency acoustic resonance and added to RAW264.7 macrophages cultured in confocal dishes. Fluorescence images were acquired using confocal laser scanning microscopy after 0, 1, 2, 4, and 8 h of incubation to assess the time-dependent cellular uptake of the nanofibers. After 8 h of co-incubation, cells were sequentially stained with Hoechst nuclear stain and LysoTracker lysosomal probe. Multichannel confocal fluorescence images were then acquired to evaluate the intracellular trafficking and lysosomal co-localization of GA@Cy5 nanofibers.

### 2.5 Colloidal stability analysis

The colloidal stability of the nanofiber complexes was evaluated by dynamic light scattering (DLS) using an Anton Paar particle size analyzer. The prepared GA@BA and GA@Ast nanofiber suspensions were diluted 10 times with ultrapure water, placed in standard zeta potential cells, and measured at 25°C. Each sample was analyzed in triplicate, with results expressed as mean ± standard deviation (SD). A zeta potential absolute value greater than 30 mV typically indicates good physical stability of the system.

### 2.6 Determination of apparent drug solubility

The equilibrium solubility method was used to evaluate the enhancement of the apparent solubility of BA and Ast. Excessive amounts of free BA, Ast, GA@BA complex, and GA@Ast complex were accurately weighed and separately dispersed in ultrapure water, followed by shaking in a constant temperature metal bath at 37°C for 24 h to reach dissolution equilibrium. Then, the suspension was centrifuged at 14,000 rpm for 15 min, and the supernatant was filtered through a 0.22 μm microporous membrane. The absorbance was measured at the maximum absorption wavelengths of BA and Ast using a UV-Vis spectrophotometer, and the drug concentrations were calculated based on pre-established standard curves.

### 2.7 Nanofiber morphology characterization

The microstructure of the nanofibers was observed using cryogenic transmission electron microscopy (Cryo-TEM, FEI Talos F200C). A 5 μL aliquot of the prepared sample was applied to a glow-discharged ultrathin carbon film-supported porous copper grid. Excess liquid was blotted away with filter paper to form a thin liquid film, and the grid was rapidly vitrified by plunging into liquid ethane. The frozen sample was transferred to a pre-cooled sample holder for observation and image acquisition at −175°C.

### 2.8 Ca^2+^-responsive gelation characterization

The gelation behavior was evaluated using both the inverted vial test and rheological measurements. Samples containing Ca^2+^ and Ca^2+^-free controls were prepared as described above. Gel formation was visually observed. Simultaneously, amplitude sweep tests (strain range 0.1% - 100%) were performed using a rotational rheometer (TA Instruments DHR-3) with a plate-plate geometry at 25°C to determine the linear viscoelastic region and the mechanical strength of the gels.

### 2.9 Fabrication of GA@BA&Ast composite microneedles

Microneedle arrays were fabricated using a template method combined with vacuum centrifugal casting. First, GA@BA hydrogel and GA@Ast hydrogel were thoroughly mixed with an 80 mg×mL^−1^ HA solution at a ratio of 1:4 for each, followed by centrifugal degassing. The resulting mixture slurry was then precisely cast into a PDMS microneedle mold. The mold was placed in a vacuum centrifuge (5000 rpm, 10 min) to ensure complete filling of the needle tip cavities. After removing excess slurry from the mold surface, a layer of pure HA solution was applied as the backing layer. Finally, the construct was dried overnight at 25°C under controlled humidity (<30% RH) to obtain the complete microneedle patch.

### 2.10 Characterization of morphology and structure of microneedles

The physical morphology and internal structure of the microneedles were systematically characterized using multiple techniques. Optical Microscopy: the overall morphology and tip integrity of the microneedle arrays were examined using a digital microscope; Scanning Electron Microscopy (SEM): microneedle samples were mounted on stubs and sputter-coated with gold (sputtering current 15 mA, time 60 s). Their surface morphology was observed using a Hitachi field emission scanning electron microscope at an accelerating voltage of 5 kV. Confocal Laser Scanning Microscopy (CLSM): GA@BA&Ast microneedles loaded with Cy5 fluorescent dye were placed on glass slides and scanned using an FV3000 laser scanning confocal microscope to confirm the uniform distribution of the drug within the needle tips.

### 2.11 Mechanical property testing

The mechanical strength of the microneedles was evaluated using an electronic universal testing machine. A microneedle (tip facing upward) was fixed on the sample stage. A pressure sensor was used to apply a vertical force at a test speed of 1.0 mm×min^−1^ until needle fracture occurred. The force-displacement curve was recorded, and the fracture energy of the microneedle was quantified by calculating the area under the curve (AUC).

### 2.12 Osmolality-responsive drug release and mechanistic analysis

Artificial saliva with osmolalities of 150, 300, and 600 mOsm/L was prepared as the release medium. Gradient osmolality artificial saliva was prepared according to ISO/TR10271 standard, with detailed ion composition and experimental parameters provided in Tables S1 and S2, respectively. GA@BA&Ast microneedles were separately incubated in the respective media for 20 min. After incubation, incompletely dissolved HA matrix residues were collected, washed three times with deionized water, and freeze-dried. Attenuated total reflectance Fourier-transform infrared spectroscopy (ATR-FTIR) and X-ray photoelectron spectroscopy (XPS) were used to analyze changes in the crosslinked network and elemental composition of the residual matrix. The corresponding release media were collected and centrifuged, and the supernatants were analyzed using a microplate reader at the characteristic absorbance wavelengths of BA and Ast to quantify the cumulative release of both drugs.

High-resolution Na 1s spectra were acquired from the hydrogel residues and reference samples, including dried NaCl and sodium hyaluronate, or HA exposed to NaCl followed by the identical washing and freeze-drying procedure. All spectra were charge-corrected using the same reference and fitted using identical constraints. The Cl 2p region was simultaneously analyzed to assess residual NaCl.

### 2.13 Evaluation of skin penetration capacity

Freshly excised rat skin (with intact epidermis and dermis) was used as the model tissue. Cy5.5-labeled microneedles were inserted vertically into the skin tissue with constant pressure. The punctured skin samples were fixed in confocal dishes. Z-stack scanning was performed using an FV3000 laser scanning confocal microscope, starting from the tissue surface and proceeding along the Z-axis in 20 μm intervals until the fluorescent signal at the bottom of the penetration track disappeared, thereby determining the actual penetration depth of the microneedles.

### 2.14 Microneedle dissolution assessment

Dissolution experiments were conducted under conditions simulating the oral environment (37°C, relative humidity >90%). The microneedle array was pressed vertically into prepared neonatal rat dorsal skin, ensuring complete insertion. At predetermined time points (1 min, 2 min, 5 min, 10 min, and 20 min), the microneedles were carefully removed. The morphological changes and dissolution process of the tips were immediately recorded using a high-definition optical microscope, and the residual volume of the needle tips was quantitatively analyzed using ImageJ software.

### 2.15 Adhesion and retention performance of GA@BA&Ast MNs

SD rats were anesthetized by isoflurane inhalation. The prepared GA@BA&Ast microneedles were then applied to the ulcerated buccal mucosa. Gross images were acquired using a high-resolution camera at 0, 20, 40, 60, and 120 min after application to monitor the adhesion status and retention of the microneedle patches. These observations were used to assess their adhesion durability under the wet oral environment and physiological oral movements.

### 2.16 Drug release study

Agar was used to simulate the oral mucosal environment for a more accurate assessment of the burst release behavior. HA-based and chitosan-based microneedles were inserted into the agar and incubated at 37°C. At predetermined time points (5 min, 10 min, 20 min, and 30 min), the entire gel block was removed, crushed, and the released drugs were extracted using methanol. BA and Ast were quantified by liquid chromatography-mass spectrometry (LC-MS).

### 2.17 Cell culture

RAW264.7 mouse macrophages and human umbilical vein endothelial cells (HUVECs) were obtained from the School of Basic Medical Sciences, Xi'an Jiaotong University. All cells were cultured in DMEM medium supplemented with 10% fetal bovine serum and 1% penicillin-streptomycin at 37°C in a 5% CO_2_ incubator.

### 2.18 Cellular uptake

RAW264.7 cells were used as the model to evaluate drug uptake efficiency. Cells were co-incubated with free Cy5 or GA@Cy5 nanofibers (equivalent Cy5 concentration). At predetermined time points (0.5 h, 1 h, 2 h, and 4 h), intracellular fluorescence signals were dynamically observed using a Keyence BZ-X300 confocal microscope. The kinetic process resulting from the difference in uptake between the loaded (GA@Cy5) and unloaded (free Cy5) formulations was compared, and images before and after loading were continuously captured using confocal microscopy.

### 2.19 In vitro biological function assessment

*Scratch wound healing assay*: A scratch was created in a confluent monolayer of HUVECs using a sterile 200 μL pipette tip. Cells were treated with extracts from various experimental groups (PBS control, GA MNs, GA@BA MNs, GA@Ast MNs, GA@BA&Ast MNs) prepared in serum-free medium. Images were captured at 0 and 24 h using an inverted microscope, and the wound closure area was quantified.

*In vitro* angiogenesis assay: Pre-cooled Matrigel matrix was evenly coated into 96-well plates and polymerized at 37°C for 30 min. HUVECs were resuspended in serum-free medium containing different microneedle extracts and seeded onto the Matrigel. After incubation for 6-8 h, live cells were stained with Calcein-AM. Tubular structures were imaged using a confocal microscope, and the total tube length and number of branching points were analyzed.

Anti-inflammatory efficacy assessment: RAW264.7 cells were stimulated with lipopolysaccharide (LPS, 1 μg×mL^−1^) for 12 h to establish an inflammation model. The medium was then replaced with fresh medium containing different microneedle extracts for another 6 h. Cell culture supernatants were collected, and the concentrations of IL-6 and TNF-α were measured using ELISA kits according to the manufacturer's instructions.

ROS scavenging assay: LPS-pre-stimulated RAW264.7 cells were treated with different microneedle extracts for 6 h. The medium was then replaced with serum-free medium containing 20 μM DCFH-DA and incubated in the dark for 30 min. After PBS washing, the fluorescence intensity was immediately measured using a microplate reader (excitation wavelength 488 nm, emission wavelength 525 nm), and the ROS scavenging rate was calculated.

### 2.20 In vivo therapeutic efficacy and safety evaluation

*Oral ulcer model establishment and treatment*: Standardized oral ulcers were induced on the rat oral mucosa using the acetic acid chemical burn method. After successful modeling, rats were randomly divided into 4 groups (n ≥ 3) and treated with: PBS (negative control), blank HA microneedles, GA@BA microneedles, and GA@BA&Ast microneedles. Ulcer area changes were recorded daily over a 7-day treatment period.

*Histological and immunohistochemical analysis*: After euthanasia, ulcer and surrounding tissues were collected, fixed in 4% paraformaldehyde, embedded in paraffin, and sectioned. Sections were subjected to H&E staining, Masson's trichrome staining (to assess collagen deposition), and immunohistochemical staining for CD31 (marking vascular endothelium), VEGF (vascular endothelial growth factor), IL-6, and TNF-α. Semi-quantitative analysis of the stained sections was performed using ImageJ software.

*Preliminary systemic toxicity assessment*: At the end of the experiment, major organs (heart, liver, spleen, lung, kidney) were collected for H&E staining to observe any pathological lesions and preliminarily assess biosafety.

*Hemolysis test*: Conducted strictly following the National Standard. A 2% red blood cell suspension was prepared and co-incubated with microneedle extracts, negative control (normal saline), and positive control (ultrapure water) at 37°C for 4 h. After centrifugation, the absorbance of the supernatant at 540 nm was measured, and the hemolysis rate was calculated.

Cytocompatibility evaluation: The cytocompatibility of GA@BA&Ast microneedles was evaluated using human umbilical vein endothelial cells (HUVECs) by combining a quantitative Cell Counting Kit-8 (CCK-8) assay with qualitative live/dead fluorescence staining. For the CCK-8 assay, HUVECs were seeded in 96-well plates. When cell confluence reached approximately 80%, the medium was replaced with medium containing microneedle extracts and cells were incubated for a further 24 h. CCK-8 reagent was then added, and absorbance was measured at 450 nm using a microplate reader to calculate cell viability. For live/dead staining, HUVECs were seeded in confocal dishes at a density of 1 × 10^5^ cells per dish. After treatment with microneedle extracts for 24 h, cells were stained with calcein acetoxymethyl ester (Calcein-AM) and propidium iodide (PI). The distribution of green-fluorescent live cells and red-fluorescent dead cells was observed by confocal laser scanning microscopy to qualitatively assess cytotoxicity.

### 2.21 Ethics

All animal experiments were conducted following with the guideline and animal protocol (NO.2023-2049 and NO. 2025-0657) approved by the ethics committees of the Xi'an Jiaotong University, which complied with the “Laboratory Animal Guideline for Ethical Review of Animal Welfare” (China, 2018) and “ARRIVE Guidelines 2.0” (2020). Male Sprague-Dawley rats (weight 180-220 g) were provided by Shaanxi Pharmaceutical Medical Biotechnology Co., Ltd. and housed in an SPF environment with free access to food and water.

### 2.22 Statistical analysis

All experiments were independently repeated at least three times. Data are presented as mean ± SD. Statistical analysis was performed using GraphPad Prism 9.0 software. One-way or two-way analysis of variance (ANOVA) with Fisher's LSD posttest was used for multiple comparisons. The difference was regarded as statistically significant (*P < 0.05, **P < 0.01, ***P < 0.001, ****P < 0.0001). All image quantifications were performed using ImageJ software.

## 3 Results and discussion

### 3.1 Preparation and characterization of GA@BA and GA@Ast nanofibers

To construct uniform and stable drug-loaded nanofibers (GA@BA and GA@Ast), the optimal mass ratios of the carrier GA (Fig. 2A) to the drugs were determined to be 20:1 for GA@BA and 15:1 for GA@Ast by screening multiple ratios, respectively (Fig. S2). Cryo-electron microscopy observations in an aqueous environment confirmed the formation of stable drug-loaded nanofibers with diameters between 3 and 4 nm, lengths up to 5 μm, and an aspect ratio reaching 1250, demonstrating high uniformity (Fig. 2B–C). The absolute value of the surface zeta potential exceeded 40 mV (Fig. S3). After the formation of the drug-loaded nanofibers, the apparent solubility of BA and Ast significantly increased to 775.50 μg×mL^−1^ and 95.16 μg×mL^−1^, representing 127.5-fold and 213.7-fold enhancements, respectively (Fig. 2D–E). Upon adding a CaCl_2_ solution at a concentration of 11.1 mg×mL^−1^ to the drug-loaded nanofiber solution, a stable gel structure formed rapidly within 1 min. Scanning electron microscopy results revealed a microstructure typical of electrostatically cross-linked gels (Fig. 2F–G) [51,52]. Shear deformation mechanical tests demonstrated the gel's typical self-healing capability (Fig. 2H), and rheological results further confirmed that the material exhibits characteristic rheological properties of hydrogels (Fig. S4).

**Fig. 2:**
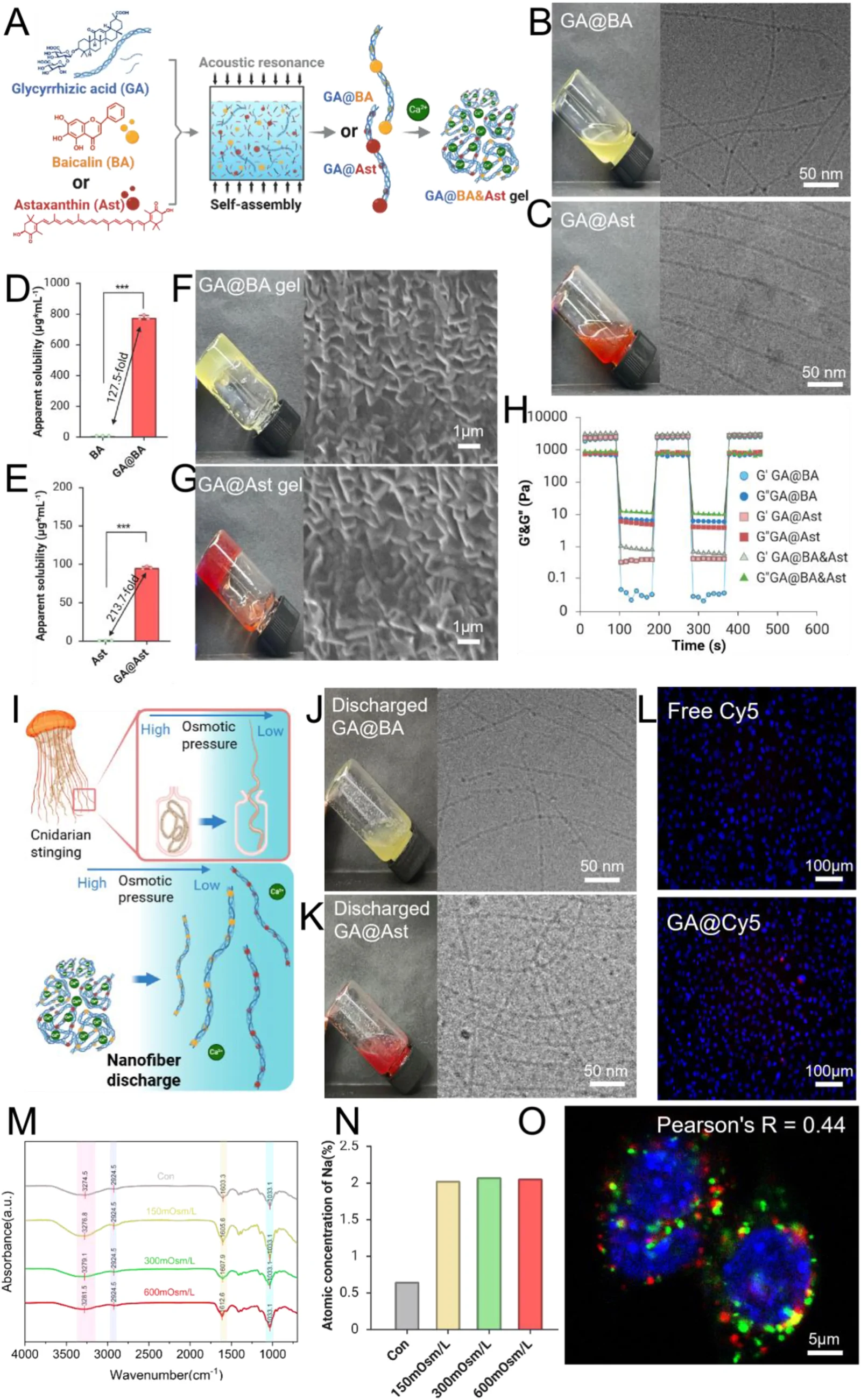
The characteristics of nanofibers (GA@BA and GA@Ast), Ca^2+^-induced gelation, and osmotic-pressure-response discharge like jellyfish stinging. (A) Schematic illustration of nanofibers preparation and hydrogel formation. (B) The representative picture (left) and Cryo-TEM image (right) of GA@BA nanofiber. (C) The representative picture (left) and Cryo-TEM image (right) of GA@Ast nanofiber. (D) Apparent solubility of free BA and GA@BA. (E) Apparent solubility of free Ast and GA@Ast. (F) The representative picture (left) and SEM image (right) of GA@BA gel. (G) The representative picture (left) and SEM image (right) of GA@Ast gel. (H) Thixotropic experiment by continuous step strain measurement of the GA@BA gel, GA@Ast gel, and GA@BA&Ast gel. (I) Schematic illustration of osmotic-pressure-response discharge of nanofibers. (J) The representative picture (left) and Cryo-EM image (right) of discharged GA@BA. (K) The representative picture (left) and Cryo-EM image (right) of discharged GA@Ast. (L) The representative cellular uptake fluorescence images of RAW264.7 cells with the treatment of free Cy5 or discharged GA@Cy5 for 4 h. (M) ATR-FTIR spectra of the hydrogel residues after exposure to artificial saliva with different osmolalities (Con (0 mOsm/L), 150, 300, 600 mOsm/L). (N) XPS analysis of hydrogel residues after exposure to media with different osmolalities, including the surface atomic percentage of Na, high-resolution Na 1s spectra. The Na 1s spectra were compared with identically processed NaCl reference samples. (O) Representative confocal fluorescence image showing partial co-localization of released GA@Cy5 nanofibers with lysosomes in RAW264.7 macrophages after incubation, with a Pearson's correlation coefficient of 0.44. Data are presented as the means ± SD. ns: not significant, ***p < 0.001.

The gel structure we developed possesses the ability, analogous to jellyfish stinging cells, to respond to environmental osmotic pressure and ionic conditions by releasing its functional components. This enables the responsive release of drug-loaded nanofibers in ulcerated epithelial tissue (Fig. 2I). Under simulated osmotic pressure and ionic conditions *in vitro*, the gel noticeably thinned, exhibiting characteristics of a solution. Cryo-electron microscopy confirmed that the released GA@BA and GA@Ast nanofibers remained intact and fully extended (Fig. 2J–K).

To investigate the potential mechanism underlying osmolality-responsive dissociation and release, samples exposed to media with osmolalities ranging from 0 to 600 mOsm/L were analyzed by ATR-FTIR, XPS, and drug-release assays. ATR-FTIR spectra showed osmolality-dependent shifts in the hydroxyl- and carboxyl-associated bands of HA, whereas no obvious change was observed in the glycosidic-bond-associated band (Fig. 2M). These spectral changes suggest alterations in local hydrogen-bonding interactions and the chemical environment surrounding HA carboxyl groups, while no substantial change in the HA backbone was detected under the tested conditions.

High-resolution XPS Na 1s spectra showed that the main Na 1s peak in the HA matrix residues after osmotic treatment was located at 1071.5-1072.0 eV. Compared with the NaCl reference sample subjected to the same processing procedure, this signal exhibited a reproducible binding-energy shift consistent with residual NaCl was detected (Fig. 2N and Fig. S5). Together with the FTIR changes in the carboxyl-associated bands, these results support the presence of Na^+^ in a carboxylate-associated ionic interaction/coordination environment within the HA matrix, rather than predominantly as residual free NaCl crystals.

Notably, Na-related signals reached a plateau at osmolalities of 150 mOsm/L and above, whereas drug release continued to increase with increasing osmolality. These findings suggest that Na^+^-related interactions may contribute to the initial rearrangement of the network, while the further release enhancement under hyperosmotic conditions may additionally involve network hydration, swelling, and diffusion processes.

Using GA@Cy5 as a model for the released nanofibers, cellular uptake experiments showed enhanced intracellular fluorescence compared with free Cy5 treatment (Fig. 2L). Time-dependent uptake and intracellular trafficking analyses further showed that Cy5 fluorescence in RAW264.7 macrophages increased over the 0–8 h incubation period (Fig. S6). Dual-channel fluorescence imaging revealed partial overlap between Cy5-labeled nanofibers and lysosomal signals (Fig. 2O and Fig. S7), with a Pearson's correlation coefficient of 0.44. These findings suggest that lysosomal trafficking may be involved after nanofiber internalization; however, the specific endocytic pathway and the occurrence of lysosomal escape require further investigation.

### 3.2 Fabrication and characterization of GA@BA&Ast MNs

GA@BA&Ast MNs were prepared in a PDMS mold via calcium ion-induced gelation followed by a dehydration process, using a solution containing GA@BA, GA@Ast, and HA at a mass ratio of 1:1:4 (Fig. 3A) [53]. The microneedle system has base dimensions of 10 mm × 10 mm, featuring a 15 × 15 array of microneedles uniformly distributed across the base. The center-to-center spacing between adjacent microneedles is 600 μm, with each individual microneedle having a base width of 300 μm and a height of 600 μm. The height was determined based on the anatomical characteristics of the oral epithelium, ensuring penetration of the oral stratum corneum barrier without causing excessive damage to deeper tissues (Fig. 3B). The drug loading process was simulated using Cy5, and fluorescence microscopy imaging showed uniform distribution of the simulated drug within the microneedles (Fig. 3C) [54]. Scanning electron microscopy images of the microneedles revealed good consistency, intact tips, orderly arrangement, and a surface roughness characteristic of the gel network (Fig. 3D) [55,56]. Optical microscopy likewise showed uniform and intact needle morphology (Fig. S8). These results indicate that GA@BA&Ast MNs can form a stable, controllable, and reproducible microneedle system fabricated using the mold-based method. The mechanical properties and responsive drug release profile require further investigation.

**Fig. 3:**
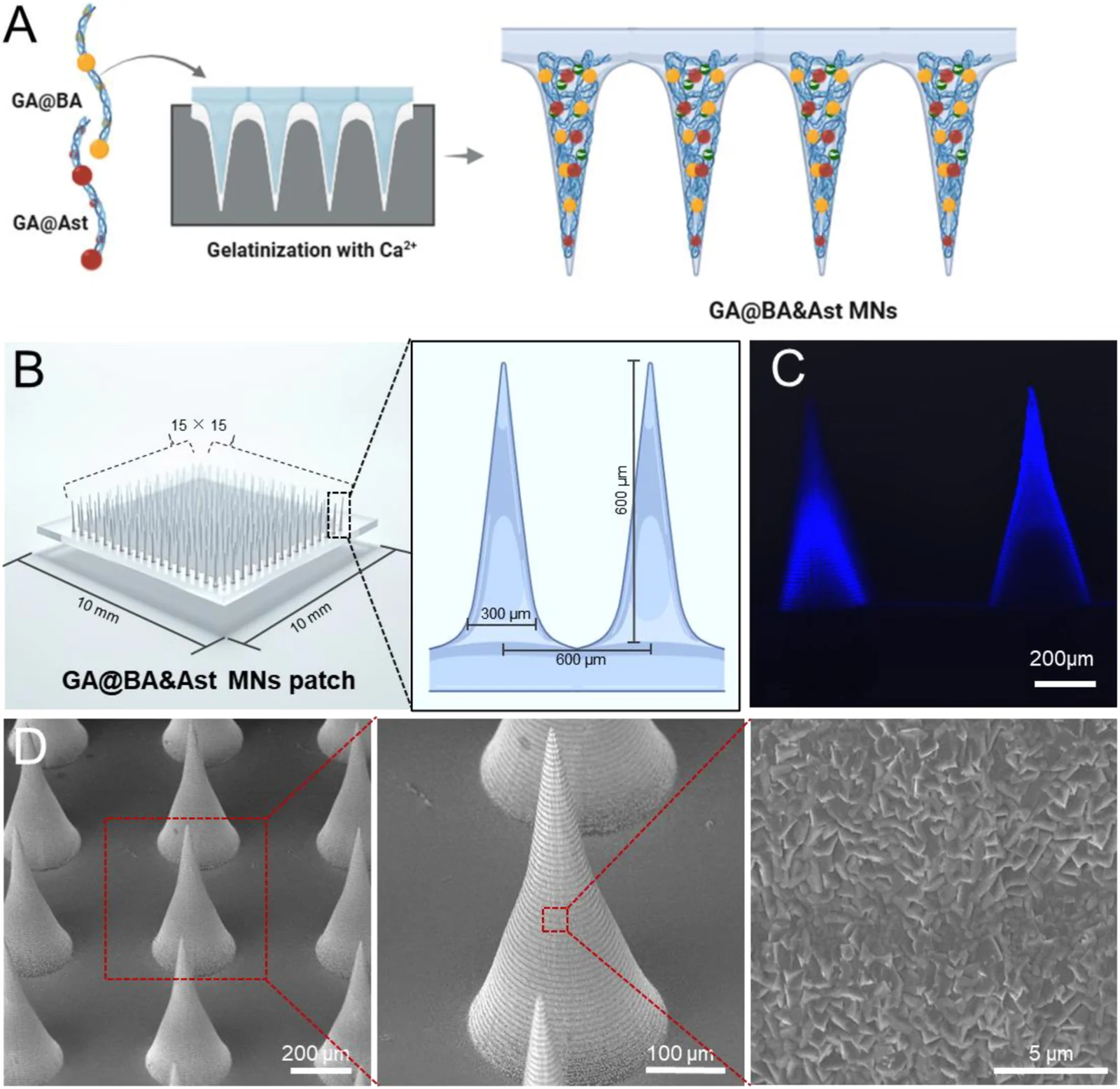
GA@BA&Ast MNs were constructed by template method. (A) Schematic illustration of GA@BA&Ast MNs preparation. (B) Dimensional schematic of the GA@BA&Ast microneedle patch. (C) Confocal image of Cy5-labeled GA@BA&Ast MNs tips. (D) The representative SEM images of GA@BA&Ast MNs.

### 3.3 Responsive immediate-release of GA@BA&Ast MNs

To verify whether the as-fabricated MNs possessed sufficient mechanical strength to penetrate the epithelium and could rapidly disintegrate in the microenvironment of ulcer tissue to release drug-loaded nanofibers, which were then quickly taken up by cells to achieve precise localized drug delivery (Fig. 4A), a series of experiments was conducted. The confocal Z-axis scanning images after fluorescently labeled GA@BA&Ast MNs penetrating rat epithelial tissues visually demonstrated that the MNs could deliver drugs to at least 220 μm depth post insertion (Fig. 4B) [57], effectively transporting the drug to the deep epithelial layers and overcoming the biological barrier. The quantitative analysis of force-displacement curves and AUC revealed the underlying reason: compared with HA gel MNs, GA@BA&Ast MNs exhibited a 1.56-fold increase in mechanical strength within the displacement range of 300 μm due to the denser electrostatic network (Fig. 4C–D) [58]. This strength was significantly higher than the minimum force required for epidermal penetration (0.15 N), supporting the ability of the microneedles to effectively penetrate the epithelial barrier. *In vivo* adhesion observations on the buccal mucosa of SD rats showed that the backing layer and/or residual matrix of the microneedle patch remained visibly attached to the mucosal surface without apparent detachment for up to 2 h after application (Fig. S9). These findings support the retention stability of the patch in the wet oral environment and suggest that it can remain in place during the period required for rapid drug release.

**Fig. 4:**
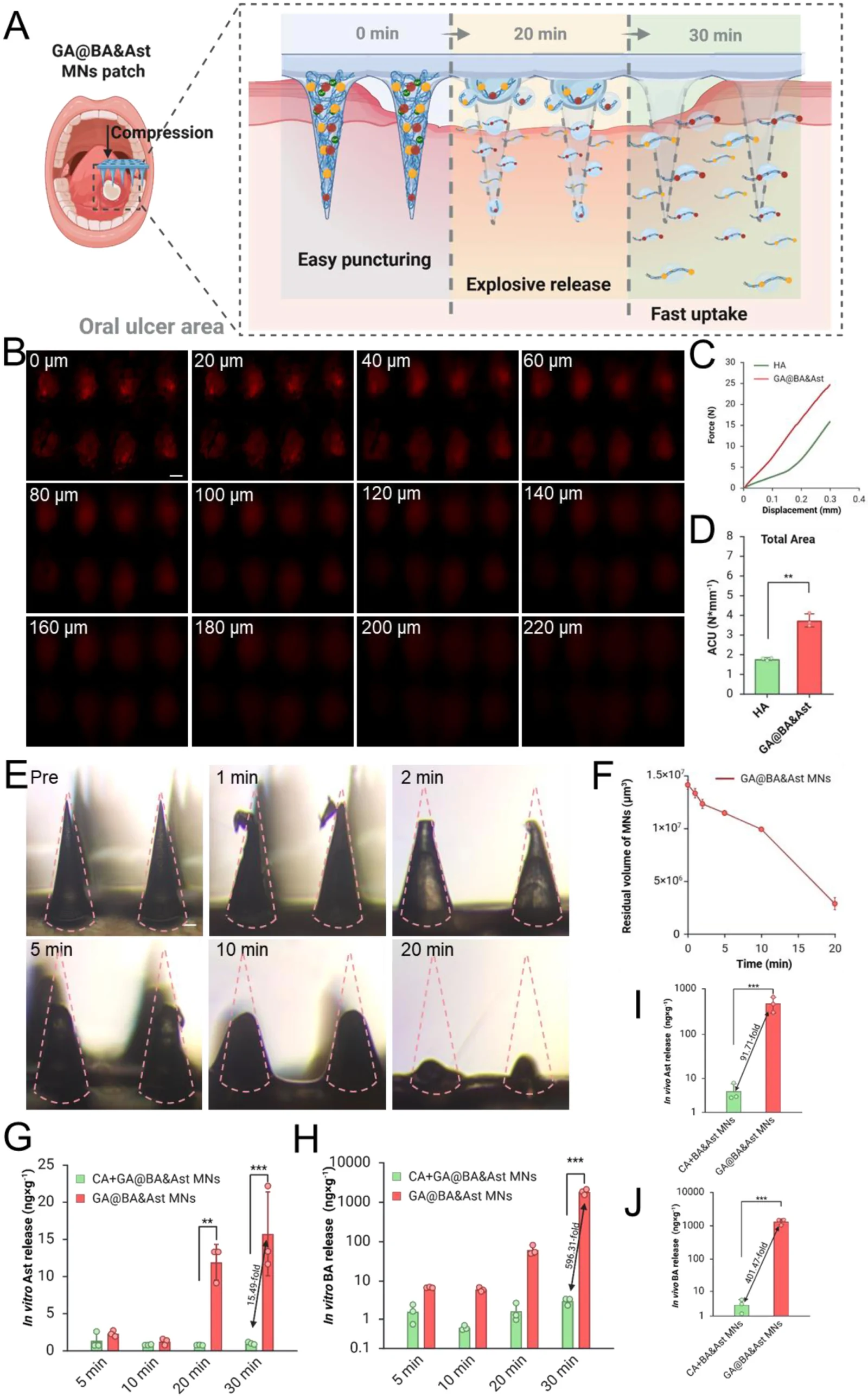
Responsive immediate-release and efficient delivery of GA@BA&Ast MNs. (A) Schematic diagram of the drug release process of GA@BA&Ast composite microneedles in the oral ulcer area. (B) Representative confocal scanning fluorescence image of rat epidermal tissue penetrated by Cy5.5-labeled GA@BA&Ast composite microneedles. Scale bar: 50 μm. (C) Force-displacement curves of HA microneedles and GA@BA&Ast composite microneedles. (D) Quantitative analysis of the area under the curve from the force-displacement curves in (c). (E) Dynamic dissolution images of GA@BA&Ast composite microneedle tips. (F) Quantitative analysis of the dynamic dissolution process in (e). (G) Quantitative analysis of Ast release during the dissolution of GA@BA&Ast composite microneedles and CA + GA@BA&Ast composite microneedles. (H) Quantitative analysis of BA release during the dissolution of GA@BA&Ast composite microneedles and CA + GA@BA&Ast composite microneedles. (I) Quantitative analysis of Ast uptake in oral tissues *in vivo* after 30 min treatment with GA@BA&Ast composite microneedles or CA + GA@BA&Ast composite microneedles. (J) Quantitative analysis of BA uptake in oral tissues *in vivo* after 30 min treatment with GA@BA&Ast composite microneedles or CA + GA@BA&Ast composite microneedles. Data are presented as the means ± SD. ns: not significant, **p < 0.01, ***p < 0.001.

The kinetic dissolution process of GA@BA&Ast MNs was evaluated *in vitro* using rat skin to simulate the oral cavity environment. The results showed that the needle tips rapidly dissociated and disappeared within 2 min, while the base of the needles significantly swelled and deformed. Subsequently, the microneedles quickly disintegrated within 20 min with minimal remnants left behind, resulting in a burst release of drug-loaded nanofibers (Fig. 4E) [59]. Quantitative analysis indicated that more than 85% of the volume of the microneedles had been decomposed within 20 min (Fig. 4F). To further investigate whether the disappearance of the structure of the microneedles led to effective release of the drug-loaded nanofibers, we verified this in an agar-simulated oral environment. Quantitative drug analysis by LC–MS revealed that the release efficiency of GA@BA&Ast MNs increased dramatically compared to chitosan microneedles. The release rates of, respectively (Fig. 4G–H). After drug release, subsequent cellular uptake is required to meet the requirements for precision drug delivery. Therefore, we conducted experiments inserting microneedles into the oral mucosa of rats to monitor whether the rapidly released drug-loaded nanofibers could be rapidly absorbed by ulcer tissues after insertion. The results showed that the total amount of drugs taken up from GA@BA&Ast MNs was significantly higher than that from chitosan microneedles. Within 20 min, most of the released Ast and BA were rapidly absorbed. At 30 min, the absolute amounts of Ast and BA taken up had increased by 91.71-fold and 401.47-fold, respectively, compared with those of the chitosan microneedles (Fig. 4I–J). This confirmed that the design goals of rapid drug release and efficient uptake were achieved.

### 3.4 Epithelial repair and antioxidant properties of GA@BA&Ast MNs

The abnormal inflammatory microenvironment in oral ulcer seriously inhibited the epithelial tissue repair at the ulcer site, resulting in poor adhesion of mucosal cells and delayed healing. Therefore, it is very important for ulcer treatment that drug-loaded nanofibers released from GA@BA&Ast MNs can promote rapid epithelial recovery. We first evaluated the pharmacological effects of BA and Ast at different mass ratios. The concentration settings of Ast and BA for synergistic ratio screening are detailed in Table S3. As the tested dual-drug formulations showed comparable activity across the evaluated endpoints, a 1:1 BA/Ast mass ratio was selected as a balanced formulation for subsequent experiments (Fig. S10). Scratch-wound and angiogenesis assays were then performed to evaluate the pro-repair effects of the released nanofibers on HUVECs. Both GA@BA and GA@Ast released from GA@BA&Ast MNs significantly promoted cell migration, with the GA@BA&Ast treatment achieving a scratch-wound closure rate of 87.8% within 24 h (Fig. 5A–C) [60]. This effect was stronger than that of Ast-free control group and significantly better than that of GA@Ast alone (56.4%). This shows that Ast is the main factor promoting epithelial repair, while the complementary effects of GA and BA provides additional repair. According to previously reported mechanisms, this enhancement may be due to more abundant carbon sources and reducing components, thus providing greater improvement.

**Fig. 5:**
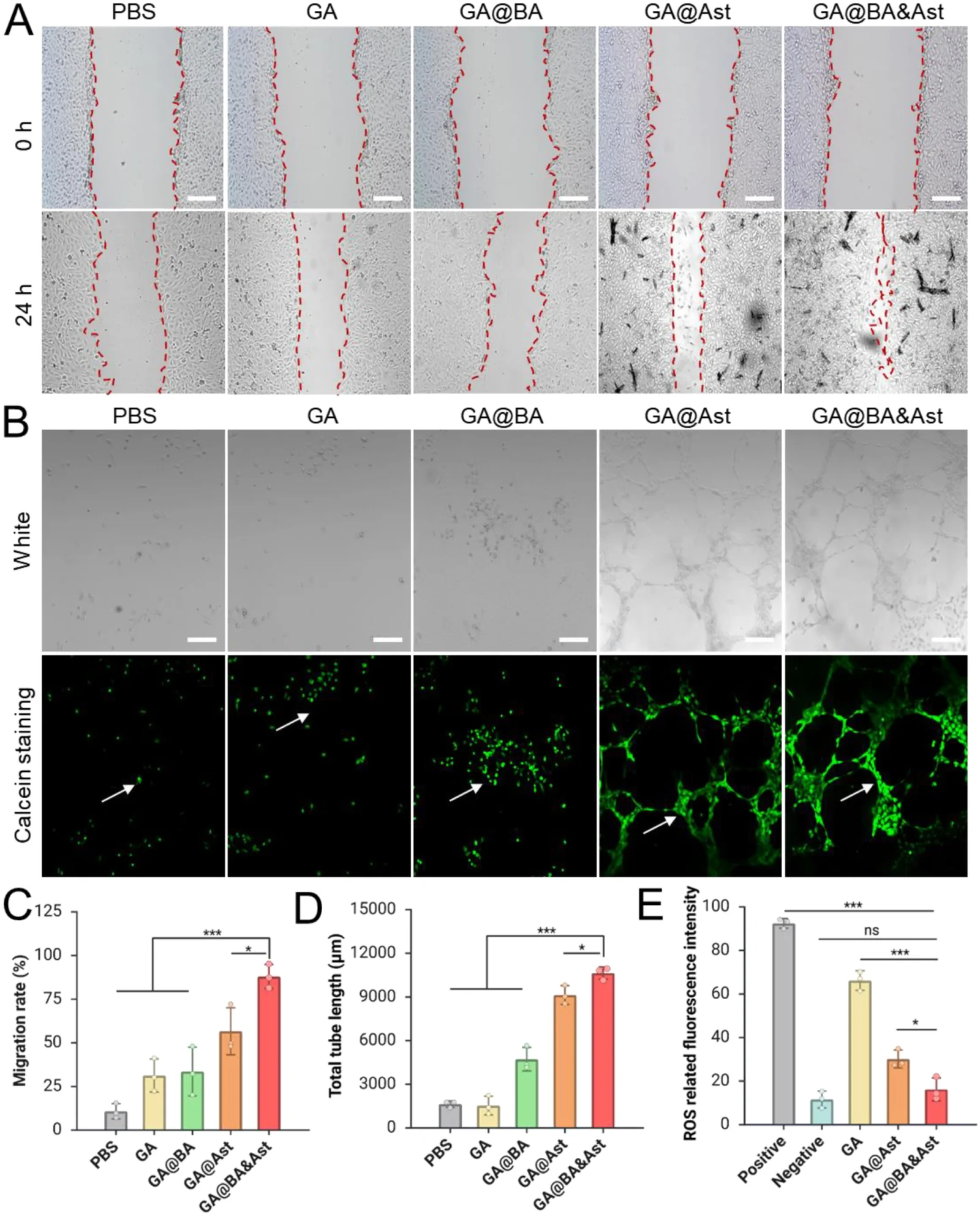
Epithelial repair and antioxidant properties of GA@BA&Ast MNs. (A) Representative images of HUVEC cell migration assays after 24-h treatment with PBS, GA, GA@BA, GA@Ast, or GA@BA&Ast. Scale bar: 200 μm. (B) Representative fluorescence images of calcein-labeled HUVEC tubular structure formation after 8-h treatment with PBS, GA, GA@BA, GA@Ast, or GA@BA&Ast. Scale bar: 200 μm. (C) Quantitative analysis of HUVEC cell migration assays. (D) Quantitative analysis of tubular network formation. (E) Quantitative analysis of ROS clearance rate. Data are presented as mean ± SD. ns: not significant, *p < 0.05, ***p < 0.001.

Revascularization is also a key factor for the recovery of full function and prevention of recurrence during ulcer healing. Therefore, we used HUVECs to take up drug-loaded nanofibers released from MNs and analyzed their effect on promoting angiogenesis with quantitative analysis (Fig. 5B–D) [61]. Both GA@BA&Ast and GA@Ast containing Ast significantly promoted the formation of tubular structures by HUVECs, resulting in clearer, more complete, and orderly connections. Among them, GA@BA&Ast again showed a more significant pro-angiogenic effect than GA@Ast due to the complementary effects of GA and BA. The drug-loaded GA@BA&Ast nanofibers released from MNs exhibited excellent abilities to promote cell migration and angiogenesis. Compared with the effects of Ast or BA alone, efficient release and rapid absorption of Ast are the keys to the observed efficacy. The mechanism of action relies on the powerful reducing ability of Ast to scavenge excessive oxidative stress. To verify the difference in ROS scavenging efficiency, LPS was used to induce cellular oxidative stress, which was then alleviated by drug-loaded nanofibers released from MNs. Benefiting from sufficient release and rapid uptake, GA@BA&Ast nanofibers exhibited the most significant ROS scavenging ability, showing a trend consistent with their repair efficacy (Fig. 5E). These results reflect the superior antioxidant capacity and fast release/absorption profile of drug-loaded GA@BA&Ast nanofibers released from MNs, indicating their obvious potential to promote epithelial repair in oral ulcers.

### 3.5 Bacteriostasis and anti-inflammatory performance of GA@BA&Ast MNs

During the healing of oral ulcer, bacterial invasion at the wound site can also hinder the repair process. BA was specifically designed to kill bacteria, thereby reducing excessive inflammation and oxidative stress caused by bacteria. Accordingly, we evaluated the antibacterial and anti-inflammatory effects of responsively released drug-loaded nanofibers from GA@BA&Ast MNs. The plate coating assay using *E. coli* and *S. aureus* confirmed the antibacterial efficacy: GA@BA&Ast effectively killed and inhibited both pathogens (Fig. 6A–C) [62], which is consistent with the antibacterial potency of GA@BA. In contrast, neither HA nor GA alone showed significant inhibitory effects, confirming that GA@BA is the core antibacterial component. In LPS-activated RAW264.7 macrophages, GA@BA&Ast treatment produced the greatest reduction in the pro-inflammatory cytokines IL-6 and TNF-α among the tested groups (Fig. 6D–E). Both GA@BA and GA@Ast also reduced cytokine expression, indicating that BA- and Ast-containing nanofibers each contribute to the anti-inflammatory activity of the combined formulation.

**Fig. 6:**
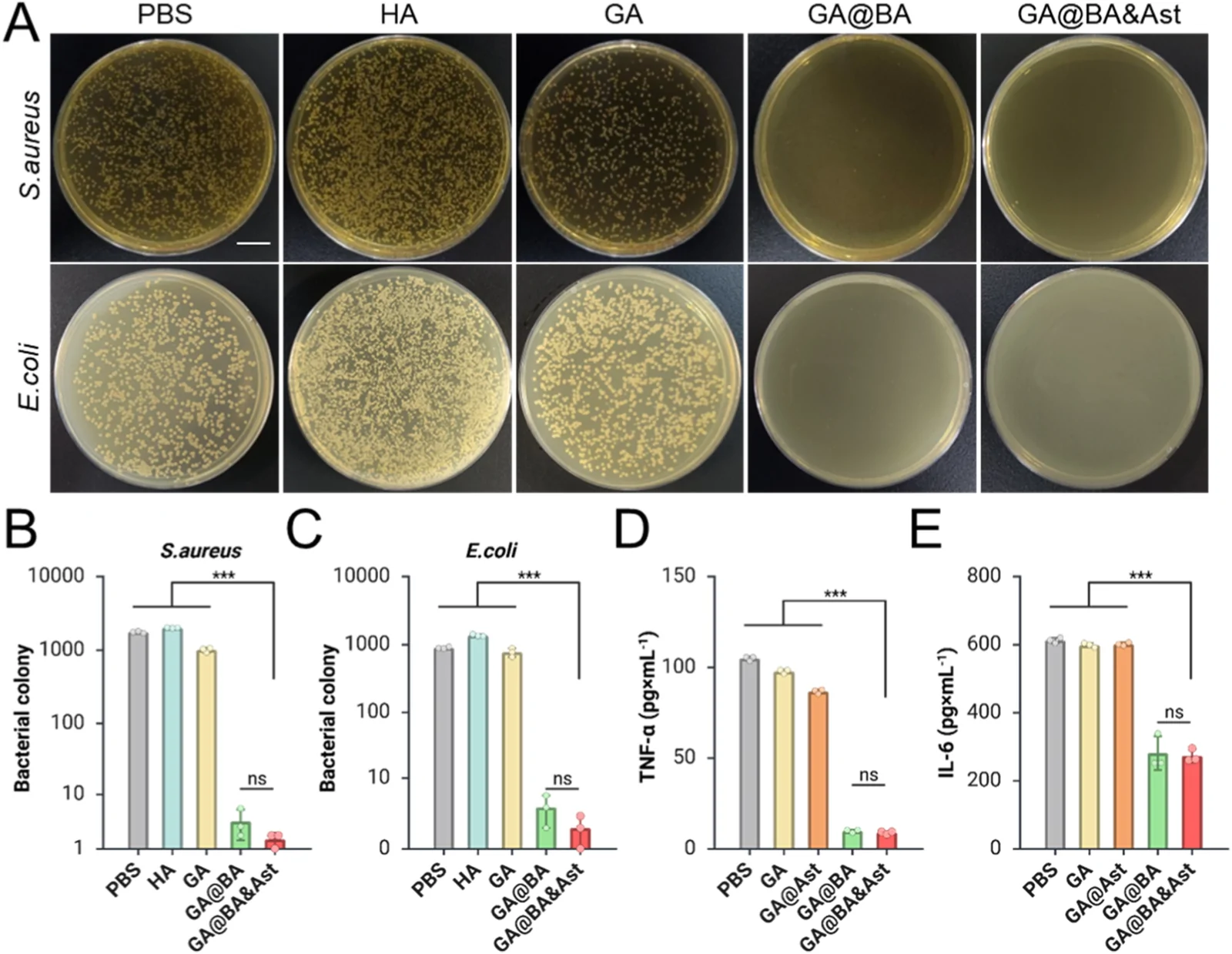
Bacteriostatic and anti-inflammatory performance of discharged GA@BA&Ast. (A) Bacteriostatic plating results for *S. aureus* and *E. coli* post-treatment by PBS, HA, GA, GA@BA, or GA@BA&Ast for 12 h. Scale bar: 1 cm. (B,C) Quantitative analysis of Bacteriostatic efficiency for *S.aureus* and *E. coli* post-treatment by PBS, HA, GA, GA@BA, or GA@BA&Ast for 12 h. The inflammatory cytokines levels of TNF-α (D) and IL-6 (E) in different treatment groups (PBS, GA, GA@Ast, GA@BA, or GA@BA&Ast) were detected by ELISA (n = 3). Data are presented as the means ± SD. ns: not significant, ***p < 0.001.

Collectively, after responsive release from the MNs, GA@BA and GA@Ast provided complementary therapeutic effects. GA@BA contributed predominantly to the antibacterial activity, whereas GA@Ast contributed primarily to antioxidant and pro-repair effects, including enhanced angiogenesis. Their combined delivery was associated with enhanced anti-inflammatory activity, improved overall therapeutic outcomes, and accelerated oral-ulcer healing.

### 3.6 Oral ulcer therapy

Building on the demonstrated rapid responsive release and efficient cellular uptake of BA and Ast from GA@BA&Ast MNs, which enable precise and efficient drug delivery to oral ulcer sites, as well as the compelling *in vitro* evidence for the superior ulcer-healing ability of the delivered GA@BA and GA@Ast nanofibers, we established a rat model of oral ulceration (Fig. 7A) [63] to evaluate the *in vivo* therapeutic efficacy of the microneedles. The change in ulcerated area was monitored and quantitatively analyzed during the 7-day treatment period (Fig. 7B–D). A significantly higher wound closure area of 77.3% was achieved by the group treated with GA@BA&Ast MNs compared to only 25.5% natural healing in untreated group. The blank MNs (without drugs) and GA@BA MNs groups also showed limited repair efficiency with wound closure areas of 34.9% and 54.3%, respectively. Macroscopically, the GA@BA&Ast MNs group exhibited a markedly enhanced rate of ulcer healing (Fig. 7C) [64]. Moreover, the ulcerated tissue treated with GA@BA&Ast MNs displayed restored redness, indicating that the healed epithelium underwent rapid revascularization, which did not occur in other control groups. Oral ulcer healing generally progresses through four stages: acute inflammation, granulation tissue formation, re-epithelialization, and maturation/remodeling. Based on the observed macroscopic characteristics, the ulcers treated with complementary fast-releasing GA@BA&Ast MNs were likely advanced to the maturation/remodeling stage. In contrast, the untreated group remained primarily at the stage of granulation tissue formation, while the GA@BA MNs group, which effectively inhibited bacterial growth, had progressed to the re-epithelialization stage.

**Fig. 7:**
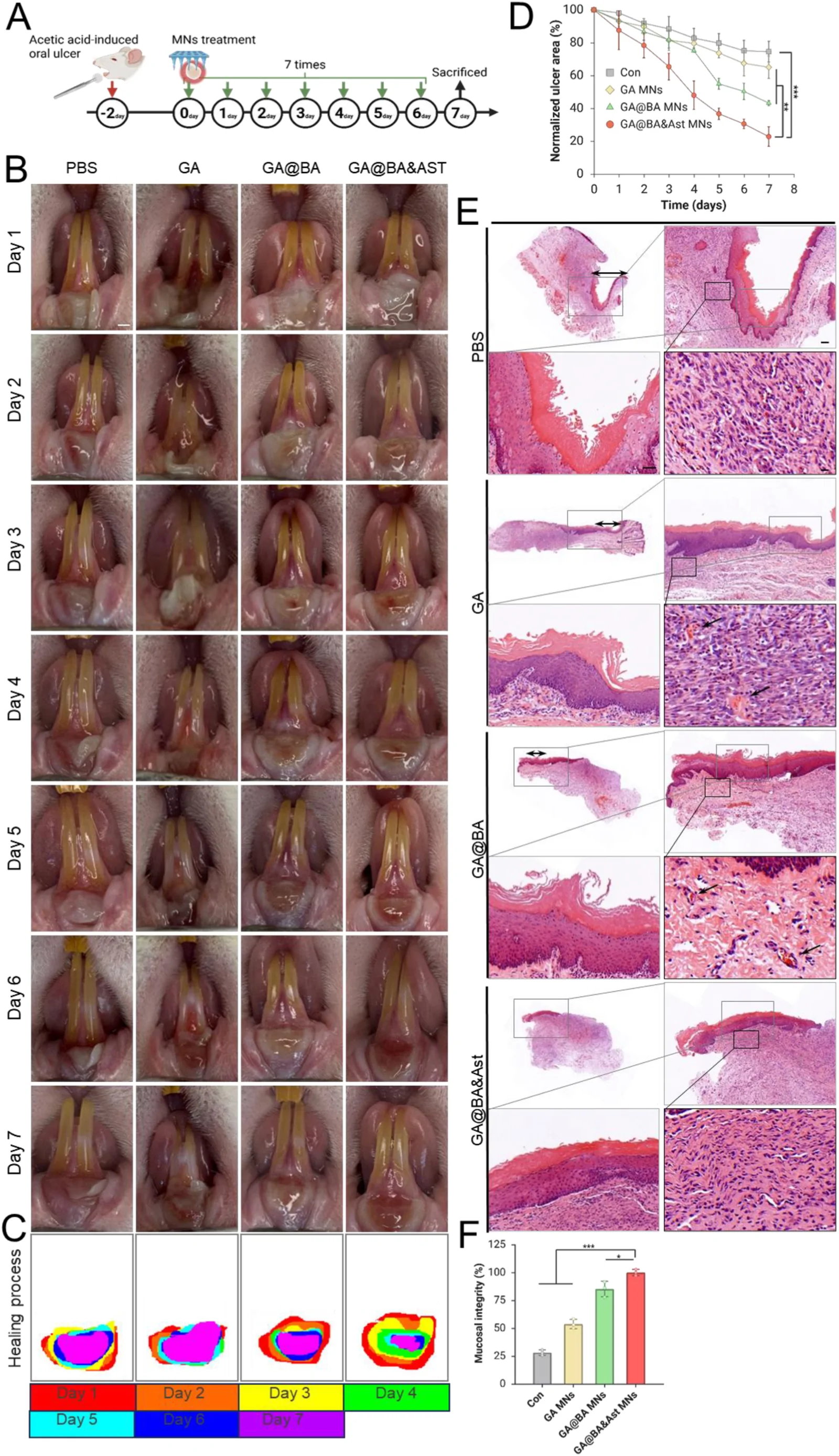
*In vivo* therapeutic efficacy of GA@BA&Ast MNs for oral ulcer. (A) A scheme of the experimental procedure used to treat rats with HAc-induced oral ulcers. (B) Sequential digital images showing the healing of ulcers treated with PBS, GA, GA@BA, and GA@BA&Ast from day 1 to day 7. (C) Schematic diagram illustrating changes in ulcer morphology over 7 days in different groups. (D) Quantitative analysis of normalized ulcer area. (E) Representative H&E staining images of oral mucosa at the site of the ulcer after various treatments. (F) Quantitative analysis of the integrity of the oral mucosal epithelium following different treatments. Data are presented as the means ± SD. ns: not significant, *p < 0.05, **p < 0.01, ***p < 0.001.

To confirm the exact healing stage, histological analysis of ulcer tissues was performed by H&E staining. The untreated group showed no typical neovascularization but only initial granulation tissue growth with a high level of neutrophil infiltration, indicating that it was at an early stage of granulation. Both GA MNs and GA@BA MNs groups had entered the re-epithelialization stage as evidenced by migrating stratified squamous epithelium from wound edge, active proliferation of basal cells and neovascularization. Notably, due to effective bacteria inhibition, the GA@BA MNs group exhibited significantly less inflammatory cells (such as neutrophils) infiltrating into deep tissue than the GA MNs group, which placed its healing status near the end of re-epithelialization. Consistent with macroscopic results, the GA@BA&Ast MNs group presented smooth, continuous and complete epithelial layer covering the center of wound without folding or significant inflammatory cell infiltration, which were characteristics of final maturity phase, demonstrating most effective therapeutic effect (Fig. 7E–F) [65].To evaluate the biosafety of GA@BA&Ast MNs treatment, major organs (heart, liver, spleen, lung, and kidney) were collected from treated mice (Fig. S11). H&E staining analysis revealed intact tissue architecture for all organs without inflammation, necrosis or fibrosis, suggesting good biocompatibility and no systemic toxicity. In addition, considering possible dissolution of drug loaded nanofibers into blood circulation, their hemolytic effects on red blood cells were evaluated. The hemolysis rate was below 5%, meeting the accepted criterion for hemolytic potential (Fig. S12). Live/dead staining and CCK-8 assays further showed that HUVEC viability after treatment with GA@BA&Ast MN extracts was not significantly different from that of the PBS control group, with no apparent increase in dead cells and cell viability remaining above 90% (Fig. S13). Together with the absence of evident pathological abnormalities in the major organs, these findings support the favorable *in vitro* cytocompatibility and preliminary *in vivo* biosafety of the developed microneedle formulation.

### 3.7 Tissue regeneration, inflammatory response, and angiogenesis

To determine whether the therapeutic effect of GA@BA&Ast MNs on oral ulcer was related to rapid release and efficient uptake of drug-loaded nanofibers, key proteins in ulcerated tissues associated with epithelial repair, angiogenesis and anti-inflammation pathways were analyzed. Masson's trichrome staining showed different collagen deposition patterns among treatment groups. The GA@BA&Ast MNs group had uniformly distributed and well-organized collagen fibers similar to those of normal oral mucosa. In contrast, irregular arrangement of collagen with local hyperplasia and residual erythrocyte deposition was observed in both GA@BA MNs and GA MNs groups, which indicated an intermediate healing stage with incomplete remodeling. The untreated group remained at the early stage of collagen formation. These results demonstrated that fast absorption of BA and Ast promoted regular collagen regeneration while reducing inflammation disruption (Fig. 8A–B) [66]. The expression levels of CD31 and VEGF, important indicators of angiogenesis, were significantly increased in GA@BA&Ast MNs group and VEGF was widely distributed; newly formed blood vessels also exhibited a mature and organized morphology. By comparison, delayed and less structured neovascularization with lower density and alignment of blood vessels was found in GA@BA MNs group (Fig. 8A–C-D). To explore the potential molecular mechanism underlying the pro-angiogenic effect of GA@BA&Ast MNs, the phosphorylation levels of PI3K/Akt pathway-related proteins were further assessed in rat oral-ulcer tissues. Immunohistochemical analysis showed that, compared with the model control group, the GA@BA&Ast MNs-treated group exhibited significantly increased relative expression of phosphorylated Akt (p-Akt) and phosphorylated PI3K (p-PI3K) at the ulcer site (Fig. S14). In addition, VEGF expression in this group was 47% higher than that in the model control group. These findings indicate that the pro-angiogenic effect of GA@BA&Ast MNs is associated with PI3K/Akt pathway activation and increased VEGF expression, suggesting that this pathway may contribute to neovascularization at the ulcer site [67].

**Fig. 8:**
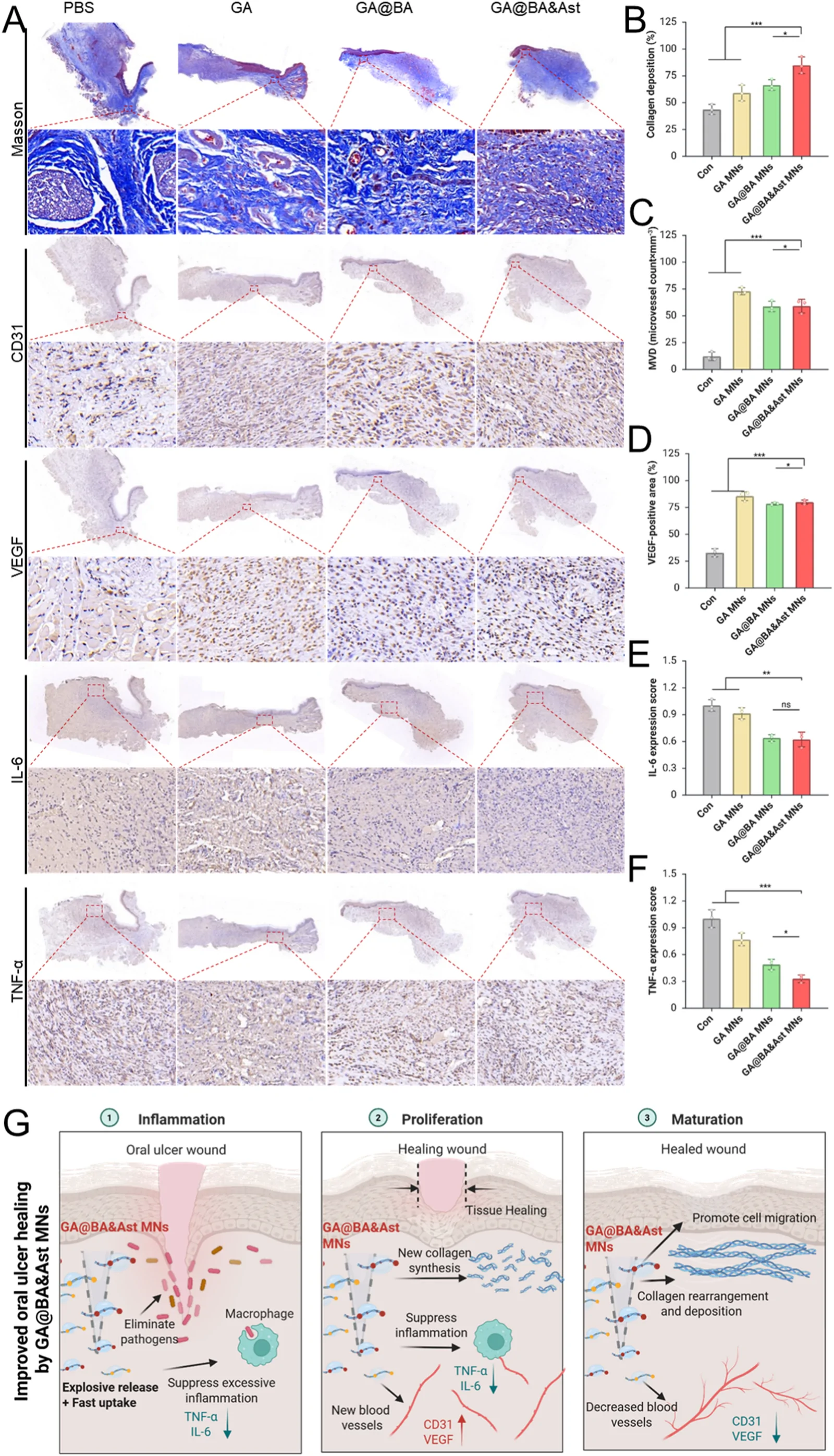
Investigation of tissue regeneration, inflammatory response, and angiogenesis at the ulcer site after different treatments. (A) Immunohistochemical (IHC) staining images of Masson, CD31, VEGF, IL-6, and TNF-α after different treatments. Scale bars: 20 μm (upper), 50 μm (lower). (B) Quantitative analysis of collagen deposition. (C) Micro-vessel density (MVD) quantification based on CD31 IHC staining. (D) Quantitative analysis of VEGF-positive staining areas. Expression levels of the anti-inflammatory cytokine IL-6 (E) and TNF-α (F). (G) Schematic Illustration of the Therapeutic Process. Data are presented as the means ± SD. ns: not significant, *p < 0.05, **p < 0.01, ***p < 0.001.

Furthermore, the levels of pro-inflammatory cytokines IL-6 and TNF-α were significantly decreased in GA@BA&Ast MNs-treated tissues, confirming that GA@BA&Ast MNs had an enhanced regulation effect on local inflammation (Fig. 8A–E-F). As shown in Fig. 8G, GA@BA&Ast MNs accelerated oral ulcer healing through a complementary multistage mechanism. In the inflammation stage, rapid release and efficient cellular uptake of active ingredients from GA@BA&Ast MNs could kill pathogenic microorganisms and inhibit macrophage overactivation to reduce the levels of pro-inflammatory cytokines (TNF-α, IL-6) and further alleviate excessive inflammatory responses. During the proliferation phase, GA@BA&Ast MNs continued to exert anti-inflammatory effects while promoting tissue repair by enhancing angiogenesis and collagen synthesis. In the maturation phase, GA@BA&Ast MNs promoted cell migration and collagen remodeling and facilitated the physiological regression of neovascularization, thereby guiding wound maturation and ultimately achieving complete ulcer healing. Overall, the superior therapeutic effects of GA@BA&Ast MNs resulted from the complementary enhancement of collagen reconstruction, angiogenesis, and anti-inflammation. The rapid drug release and high tissue uptake of GA@BA&Ast MNs created a favorable microenvironment for wound healing and accelerated oral ulcer recovery.

## 4 Conclusion

To address the challenge of effective local drug delivery in the oral mucosa due to the epithelial barrier and saliva secretion, we developed a dynamically responsive, triggerable-release microneedle platform (GA@BA&Ast MNs) inspired by the osmotic-pressure-triggered discharge mechanism of jellyfish nematocyst. The system utilized self-assembled GA with Ast or BA to form long-range ordered drug-loaded nanofibers. The designed MNs allowed rapid penetration through the epithelial barrier and released nanofibers upon response to the local osmotic environment. The released nanofibers facilitated rapid cellular uptake of drugs, overcoming short residence time of drugs and minimizing enzymatic degradation, thus enabling sustained and efficient local drug utilization for significantly improved treatment of oral ulcer and accelerated healing.

We first systematically optimized the ratios and conditions for GA@BA and GA@Ast nanofiber assembly, respectively, and identified optimal mass ratios of 20:1 and 15:1. This yielded uniform and stable nanofibers with a diameter of 3–4 nm, a length up to 5 μm, and an ultrahigh aspect ratio up to 1250. The nanofibers showed high monodispersity, an absolute value of zeta potential higher than 40 mV (Fig. S2), and anionic polymer characteristics that enabled rapid formation of a stable and self-healing gel with multivalent cations. Analogous to jellyfish nematocysts, the nanofibers exhibited osmotic and ionic responsive release: under osmotic stress or ionic competition, the gel was liquified and released intact and elongated drug-loaded nanofibers (Fig. 2). The nanostructures promoted rapid cellular uptake of drugs.

Taking advantage of this responsive release property, GA@BA&Ast MNs were fabricated using a molding method. A solution containing GA@BA, GA@Ast, and hyaluronic acid (HA) with a mass ratio of 1:1:4 was gelled and dehydrated in a PDMS mold by calcium ions to form the structure matrix with 80 mg× mL^−1^ HA. The resultant MNs showed good integrity, sharp tip, uniform drug distribution (Fig. 3), and mechanical strength that is 1.56 times than pure HA MNs, which exceeded the required force for epithelium penetration (0.15 N). Dissolution kinetics revealed that the tips of GA@BA&Ast MNs disintegrated within 2 min and more than 85% volume fraction dissolved within 20 min, releasing drug-loaded nanofibers. Release rates of Ast and BA were significantly enhanced by 15.49 and 596.31 folds compared to those from chitosan-based MNs (Fig. 4). After epithelium penetration and nanofibers release, GA@BA&Ast MNs increased absolute uptake amounts of Ast and BA by 91.71 and 401.47 folds at 30 min, respectively, confirming rapid drug release and effective uptake.

The rapidly internalized nanofibers enhanced epithelial cell migration and angiogenesis, as well as exhibited prominent ROS scavenging (Fig. 5), antibacterial and anti-inflammatory effects (Fig. 6). These complementary effects collectively contributed to the accelerated healing of oral ulcers. In a rat oral ulcer model, macroscopic observation showed that the GA@BA&Ast MNs group achieved a wound closure of 77.3%, with a significantly higher healing rate than those in the control groups (Fig. 7). The recovered pink color indicated rapid revascularization, and histological examination revealed typical re-epithelialization, active basal cell proliferation and new capillary formation, confirming that it had entered the maturation/remodeling stage. By contrast, the control groups were only at the re-epithelialization stage. Analysis of key proteins related to the pathways of epithelial repair, angiogenesis and anti-inflammation in the healed tissues consistently demonstrated that GA@BA&Ast MNs could accelerate healing while inhibiting excessive inflammation (Fig. 8). By employing a microenvironment-responsive release and uptake strategy, this system enabled efficient local treatment of oral ulcers. Many microneedle systems developed for infected skin wounds rely on antibiotic delivery, photodynamic antibacterial approaches, or sustained-release matrices. In contrast, the present study was designed for the hyperosmotic, wet, and mechanically active oral environment. The HA-Ca^2+^ coordination-crosslinked microneedle matrix underwent rapid disintegration upon exposure to ulcer-associated fluid without requiring an external stimulus, thereby facilitating rapid release of the drug-loaded nanofibers. Compared with chitosan-based microneedles, the developed system showed substantially enhanced release of BA and Ast, which may be advantageous for addressing the early inflammatory phase of oral ulcers.

At the carrier-design level, GA self-assembled nanofibers were incorporated into the dissolving microneedle matrix as a preloading platform for the hydrophobic drugs BA and Ast. This design improved the apparent aqueous solubility of both compounds and enabled osmolality-responsive drug release, with more than 85% cumulative release within 20 min.

At the therapeutic-strategy level, the formulation combines BA-associated antibacterial activity with the antioxidant, anti-inflammatory, and pro-repair effects associated with Ast. This antibiotic-free and corticosteroid-free approach may reduce reliance on conventional pharmacological treatments. In the rat oral-ulcer model, GA@BA&Ast MNs enhanced collagen deposition by 41% and VEGF expression by 47% relative to the model control group, supporting their potential to promote ulcer repair. These results highlight the potential of the responsive microneedle platform for local oral-ulcer therapy.

In conclusion, we successfully developed a universal MNs system for the targeted delivery of hydrophobic small-molecule drugs. Mimicking jellyfish nematocyst discharge, it enabled precise and efficient drug absorption after penetrating the epithelium, providing a promising universal strategy for local oral diseases such as oral ulcer, oral fibrosis, and oral cancer.

## CRediT authorship contribution statement

**Jinfan Yang:** Project administration, Funding acquisition. **Kaihong Shi:** Writing – original draft, Visualization, Software, Investigation. **Jiachen Wang:** Investigation. **Huimin Liu:** Investigation. **Yang Zhang:** Investigation. **Xiaoyu Guang:** Investigation. **Siyuan Wang:** Investigation. **Wenxi Lei:** Supervision. **Yuxiao Wang:** Supervision. **Mingqian Tan:** Supervision, Resources. **Xiaobin Ma:** Writing – review & editing, Investigation. **Hao Wu:** Writing – review & editing, Project administration, Methodology, Funding acquisition, Data curation.

## Declaration of competing interest

The authors declare that they have no known competing financial interests or personal relationships that could have appeared to influence the work reported in this paper.

## Data Availability

Data will be made available on request.
