# Supplementary material for: Hyaluronic acid-based osmolality-responsive immediate-release microneedles co-loading baicalein and astaxanthin for oral ulcer therapy
Source: Mater Today Bio. 2026 Oct 1;41:103702. doi: 10.1016/j.mtbio.2026.103702 (PMC13677995; doi:10.1016/j.mtbio.2026.103702)
Supplement: Supplementary file 1 [file mmc1.docx]

**Hyaluronic acid-based osmolality-responsive immediate-release microneedles co-loading baicalein and astaxanthin for oral ulcer therapy**

Jinfan Yang^a,b*^, Kaihong Shi^a,b,c^, Jiachen Wang^b^, Huimin Liu^b^, Yang Zhang^c^, Xiaoyu Guang^c^, Siyuan Wang^a^, Wenxi Lei^a^, Yuxiao Wang^d^, Mingqian Tan^d^, Xiaobin Ma^a,**^, Hao Wu^a,c,***^

^a^Comprehensive Breast Center, The Second Affiliated Hospital of Xi'an Jiaotong University, Xi’an 710061, China

^b^National Demonstration Center for Experimental Light Chemistry Engineering Education, Shaanxi Provincial Key Laboratory of Papermaking Technology and Specialty Paper Development, College of Bioresources Chemical and Materials Engineering, Shaanxi University of Science & Technology, Xi’an 710021, China.

^c^College School of Basic Medical Sciences, Key Laboratory of Environment and Genes Related to Diseases of Ministry of Education, Xi’an Jiaotong University, Xi’an 710061, China

^d^Academy of Food Interdisciplinary Science, School of Food Science and Technology, Dalian Polytechnic University, Qinggongyuan1, Ganjingzi District, Dalian 116034, China

* Corresponding author.

** Corresponding author.

*** Corresponding author.

E-mail addresses: yangjinfan@sust.edu.cn (J. Yang); binbinmxb@xjtu.edu.cn (X. Ma); lfshwu@xtju.edu.cn (H. Wu).

**Table. S1.** Ion composition of gradient osmolality ISO/TR10271 standard artificial salivaAll groups were prepared by proportional dilution or concentration of ISO/TR10271 standard artificial saliva stock solution, with identical ion ratios across all gradients. Con: ultrapure water control group.

| Group | Na^+^(mM) | K^+^(mM) | Ca^2+^(mM) | Cl^-^(mM) | H_2_PO_4_^-^(mM) |
| --- | --- | --- | --- | --- | --- |
| Con | 0 | 0 | 0 | 0 | 0 |
| 150mOsm/L | 6.0 | 2.7 | 2.7 | 11.5 | 2.5 |
| 300mOsm/L | 12.0 | 5.4 | 5.4 | 23.0 | 5.0 |
| 600mOsm/L | 24.0 | 10.8 | 10.8 | 46.0 | 10.0 |

**Table. S2.** Experimental parameters for gradient osmolality artificial saliva treatment.All groups were adjusted to pH 6.0 and incubated at 37°C to simulate the human oral physiological environment. osmol.: osmolality; measured osmolality was detected by freezing point osmometer, with error <3% of the designed value for all groups, within the acceptable range of instrument measurement error.

| Group | Designed osmol.(mOsm/L) | Measured osmol.(mOsm/L) | PH | Temp.(℃) | Time(min) |
| --- | --- | --- | --- | --- | --- |
| Con | 0 | 0 | 6.0 | 37 | 20 |
| 150mOsm/L | 150 | 146 | 6.0 | 37 | 20 |
| 300mOsm/L | 300 | 289 | 6.0 | 37 | 20 |
| 600mOsm/L | 600 | 617 | 6.0 | 37 | 20 |

**Table. S3.** Concentration settings of astaxanthin (Ast) and baicalein (BA) at different mass ratios.All groups were fixed at a total final drug concentration of 40μg/mL, with all ratios calculated based on mass ratio (w/w). Conc.: concentration.

| Ratio(Ast:BA) | Ast Conc.(μg/mL) | BA Conc.(μg/mL) | Total Conc.(μg/mL) |
| --- | --- | --- | --- |
| 1:0 | 40 | 0 | 40 |
| 2:1 | 26.67 | 13.33 | 40 |
| 1:1 | 20 | 20 | 40 |
| 1:2 | 13.33 | 26.67 | 40 |
| 0:1 | 0 | 40 | 40 |

**Fig. S1.** Characterization of hyaluronic acid. (A) Molecular weight distribution chart of hyaluronic acid. (B) Hydrogen nuclear magnetic resonance spectrum of hyaluronic acid.

**Fig. S2.** Screening and Characterization of GA@BA and GA@Ast Ratios. (A) Schematics of baicalein loading by glycyrrhizic acid at different ratios before and after centrifugation. (B) Schematics of astaxanthin loading by glycyrrhizic acid at different ratios before and after centrifugation.

**Fig. S3.** Stability testing of GA@BA and GA@Ast. (A) Schematic representation of Zeta potential comparisons for baicalein loaded by glycyrrhizic acid at different ratios. (B) Schematic representation of Zeta potential comparisons for astaxanthin loaded by glycyrrhizic acid at different ratios. ns: not significant, *p < 0.05, p < 0.01,*p < 0.001,****p < 0.0001.

**Fig. S4.** Characterization of GA@BA and GA@Ast Gel Properties.

**Fig. S5.** Osmotic pressure-triggered cumulative drug release. (A) Cumulative astaxanthin (Ast) release profile. (B) Cumulative baicalein (BA) release profile under gradient osmotic pressures. Both drugs show significant osmotic pressure-dependent release enhancement. ns: not significant, ****p < 0.0001.

**Fig. S6.** Time-dependent cellular uptake of Cy5-labeled GA nanofibers in RAW264.7 macrophages. (A) Confocal images at 0, 1, 2, 4 and 8 h (nuclei: blue, nanofibers: red). (B) Quantitative intracellular fluorescence intensity shows a time-dependent uptake increase. ns: not significant, ****p < 0.0001.

**Fig. S7.** Line scan analysis of lysosome co-localization. The high overlap between fluorescence intensity profiles of Cy5-labeled nanofibers (red) and lysosomes (green) confirms that internalized GA nanofibers are mainly trafficked through the lysosomal pathway.

**Fig. S8.** Optical microscopy image of the GA@BA&Ast MNs microneedle patch.

**Fig. S9.** *In vivo* mucosal adhesion stability of GA@BA&Ast microneedles on rat oral mucosa. Gross observation images at 0, 20, 40, 60 and 120 min post-implantation demonstrate the microneedles (marked by white dashed boxes) remained stably adhered to the mucosa for at least 2 hours under oral physiological conditions.

**Fig. S10.** Screening of optimal baicalein (BA)/astaxanthin (Ast) mass ratio for synergistic therapy. (A) Colony images of S. aureus (upper) and E. coli (lower) treated with PBS, single drugs (1:0, BA only; 0:1, Ast only) and combinations at different ratios. (B) Quantitative bacterial colony counts, (C) Intracellular ROS level, (D) Pro-inflammatory cytokine (TNF-α, IL-6) secretion levels after different treatments. ns: not significant, *p < 0.05, ****p < 0.0001.

**Fig. S11.** Assessment of material biocompatibility. Histotoxicity assays of the heart, liver, spleen, lungs, and kidneys. Scale bar: 200 μm.

**Fig. S12.** Hemolysis assays and hemolysis rate analysis for different treatment groups.

**Fig. S13.** Cytocompatibility evaluation of GA@BA&Ast MNs via live-dead staining. (A) Calcein-AM stained live cells (green) in PBS control and GA@BA&Ast treated groups. (B) Quantitative cell viability analysis shows no significant difference between the two groups. ns: not significant.

**Fig. S14.** Immunohistochemical analysis of PI3K/Akt pathway activation in rat oral ulcer tissues. (A) Representative staining and high-magnification views of phosphorylated Akt (p-Akt, upper) and phosphorylated PI3K (p-PI3K, lower) in tissues treated with PBS, blank GA MNs, GA@BA MNs and GA@BA&Ast MNs. (B) Quantitative relative p-Akt expression. (C) Quantitative relative p-PI3K expression. ns: not significant, ***p < 0.001, ****p < 0.0001.
